# Impact of Omnipod 5 automated insulin delivery on continuous glucose monitoring metrics and predictors of improvement in time in range

**DOI:** 10.1111/dme.70137

**Published:** 2025-09-09

**Authors:** Roland H. Stimson, Mark W. J. Strachan, Shareen Forbes, Rohana J. Wright, Scott D. Mackenzie, Gayle McRobert, Emily M. McMurray, Marcus J. Lyall, Anna R. Dover, Fraser W. Gibb

**Affiliations:** ^1^ Edinburgh Centre for Endocrinology & Diabetes NHS Lothian Edinburgh UK; ^2^ University/BHF Centre for Cardiovascular Science, Queen's Medical Research Institute University of Edinburgh Edinburgh UK

**Keywords:** continuous glucose monitoring, CSII, insulin therapy, type 1 diabetes

## Abstract

**Aims:**

This study aimed to assess the impact of the Omnipod 5 automated insulin delivery (AID) system on continuous glucose monitoring (CGM) metrics, HbA1c, and weight in a real‐world setting. Additionally, independent predictors of glycaemic response were assessed.

**Methods:**

Observational analysis of adults with type 1 diabetes using Omnipod 5 (*n* = 353). Paired data on CGM metrics (*n* = 268), HbA1c (*n* = 193), and weight (*n* = 173) were collected at baseline and compared after median of 191, 120, and 221 days, respectively. Independent predictors of TIR response (≥5%) and HbA1c (≥5 mmol/mol) were assessed.

**Results:**

Omnipod 5 use was associated with improved TIR (+16%, *p* < 0.001) and a reduction in HbA1c (−3 mmol/mol, *p* < 0.001). The greatest improvements (−7 mmol/mol, *p* < 0.001) were observed in individuals with elevated baseline HbA1c (≥58 mmol/mol). Sensor choice (Dexcom G6 vs. Freestyle Libre 2 Plus) influenced time in full auto mode (94% vs. 96%, *p* < 0.001) but did not affect the likelihood of improved TIR or HbA1c. Logistic regression identified baseline HbA1c (OR 1.24 per mmol/mol, *p* < 0.001) as the main association with improved HbA1c. Similarly, baseline TIR was associated with improvement in TIR (OR 0.83 per %, *p* < 0.001). Greater time in automation and using the lowest glucose target were also associated with improved outcomes.

**Conclusions:**

Omnipod 5 is associated with significant and sustained improvements in CGM metrics and HbA1c, particularly in individuals with higher baseline HbA1c. The results suggest the potential benefits of prioritizing AID for individuals at greatest risk of complications.

**What's new?**

**What is already known?**

- Automated insulin delivery systems, such as Omnipod 5, have demonstrated improvements in CGM metrics and HbA1c in clinical trials.
- Real‐world data on the impact on glycaemic management, weight, and the comparison between CGM systems is limited.

**What this study has found?**

- In a real‐world setting, Omnipod 5 led to significant improvements in time in range, with a median increase of 16% and reductions in time below range and HbA1c.
- Improvements in HbA1c and TIR were most pronounced in individuals with higher baseline HbA1c, particularly those previously using multiple daily injections.
- Sensor choice influenced the time spent in automation but did not independently predict the magnitude of glycaemic improvement.

**What are the implications of this study?**

- Omnipod 5 shows substantial real‐world effectiveness, especially for individuals with higher baseline HbA1c, suggesting its potential to reduce complications in those at greatest risk.
- The findings highlight the importance of maximizing time in auto mode and optimizing settings for glycaemic management.
- The study underscores the need for standardization between CGM systems to allow for accurate comparison of AID outcomes across different platforms.

## 1 INTRODUCTION

The expansion in the use of continuous glucose monitoring (CGM)^1^ and continuous subcutaneous insulin infusion (CSII)^2^ has resulted in substantial improvement in glycaemic outcomes in people living with type 1 diabetes. Despite this, only a minority of people living with type 1 diabetes achieve HbA1c <53 mmol/mol^3^ and access to diabetes technology is unevenly distributed,^4^ with particular challenges in providing therapy to those with the greatest socioeconomic disadvantage. Automated insulin delivery (AID) systems have been shown to substantially improve both CGM metrics and HbA1c.^5^ Omnipod 5 (Insulet Corp) is a tubeless AID system which was launched in the United Kingdom in 2023. Substantial improvements in CGM metrics and HbA1c have been demonstrated in the pivotal Omnipod 5 trial^6^ and in real‐world data.^7^, ^8^ The system is compatible with both the Dexcom G6 and Freestyle Libre 2 Plus CGM systems. We sought to assess the impact of Omnipod 5 upon CGM metrics, HbA1c, and weight in a real‐world context. We also assessed predictors of glycaemic response and differences in outcomes comparing Dexcom G6 and Freestyle Libre 2 Plus. This is, to the best of our knowledge, the largest real‐world assessment of changes in glycaemic metrics following the commencement of Omnipod 5.

## 2 METHODS

This was an observational assessment of outcomes in people attending Edinburgh Centre for Endocrinology & Diabetes clinics who have been commenced on the Omnipod 5 AID system. All individuals with type 1 diabetes, aged above 16 years at the start of AID, were included. People with previous AID use were excluded from the assessment. As a service evaluation of routinely collected data, this project did not require ethical approval.

Baseline CGM data (14 days) were obtained from the calendar month prior to AID commencement from either LibreView or Dexcom Clarity. Baseline CGM data were available in 310/353 (88%) people who started Omnipod 5. CGM data to assess changes in metrics were obtained from Glooko at the end of March 2025 (median duration of Omnipod 5 use was 191 days [135–452]) and paired data were available in 268/353 (76%). Glucose metrics are reported in line with the international consensus on time in range.^9^ Information on AID settings and insulin dose were also obtained from Glooko. Clinical and demographic data were obtained from SCI‐Diabetes (the Scottish national diabetes register). Baseline HbA1c was the last available measurement (median 120 days [49–248]) prior to AID commencement and was available in all but one individual. Follow‐up HbA1c was the last available HbA1c (median 228 days [125–382]) and did not include values within 60 days of AID commencement; paired HbA1c data were available in 193/353 (55%). Baseline weight was the last available measurement (median 154 days [64–266]) and was available in all but three individuals. Follow‐up weight was defined as the last available measurement (median 221 days [122–372]) and paired weight data were available in 173/353 (49%). Scottish Index of multiple deprivation (SIMD) is an area‐based measure of relative deprivation and is reported as rank (out of 6976 – 1 is most deprived) and quintile (quintile 1 is most deprived). Random plasma C‐peptide was available in 303/353 (86%) and is routinely assessed, in our centre, in all people with type 1 diabetes of at least 3 years' duration, as described previously.^10^

Data are presented as median (IQR) unless otherwise stated. Paired data were compared with Wilcoxon signed‐rank tests and unpaired data with Wilcoxon rank‐sum test. Categorical data were compared by *χ* ^2^‐test, or McNemar's test when comparing paired data. Correlations were assessed by use of Spearman's correlation coefficient. Fisher *r*‐to‐*z* transformation was used to compare correlation coefficients. Logistic regression analysis assessed independent predictors of improvement in TIR (defined as ≥5%) and HbA1c (defined as ≥5 mmol/mol). *p*‐values < 0.05 were considered statistically significant. Statistical analyses were performed using R Studio (version 2023.12.1).

## 3 RESULTS

### 3.1 Cohort characteristics and generalizability

Baseline characteristics of the cohort are described in Table 1. The only notable differences between individuals with paired CGM data and those where this was not available were younger age and shorter diabetes duration in those with no paired data; baseline HbA1c and CGM data did not differ. Of those with paired CGM data, 30% (88/298) of Omnipod 5 use was with Dexcom G6 sensors, and 70% (210/298) was with Freestyle Libre 2 plus.

**TABLE 1:** Baseline characteristics and comparison of those with paired CGM data (baseline and follow‐up) versus those with no paired CGM data.

	Total cohort (*n* = 353)^a^	Paired CGM data (*n* = 268)	No paired CGM data (*n* = 85)	*p*
Age (years)	42 (32–53)	44 (33–54)	37 (17–52)	0.011
Age at diabetes diagnosis (years)	16 (10–27)	19 (9–27)	14 (9–27)	
Duration of diabetes (years)	22 (13–32)	23 (14–33)	18 (11–28)	
Sex	Female: 56% Male: 44%	Female: 57% Male: 43%	Female: 53% Male: 47%	0.501
Weight (kg)	77.0 (66.7–88.6)	79.0 (67.9–90.0)	74.3 (65.9–82.9)	0.044
BMI (kg/m^2^)	26.4 (23.8–30.0)	26.8 (23.9–30.7)	25.7 (23.7–28.0)	0.056
Baseline HbA1c (mmol/mol)	61 (53–71)	61 (53–71)	62 (53–74)	0.711
Baseline HbA1c < 58 mmol/mol	42%	43%	40%	0.738
Insulin delivery at baseline	CSII: 71% MDI: 29%	CSII: 71% MDI: 29%	CSII: 72% MDI: 28%	0.929
C‐peptide category	<50pM: 76% ≥50pM: 24%	<50pM: 77% ≥50pM: 23%	<50pM: 72% ≥50pM: 28%	0.402
SIMD rank (1 to 6976)	4542 (2640–5996)	4593 (2635–6094)	4536 (2684–5550)	0.327
SIMD quintile category	SIMD 1 or 2: 26% SIMD 3–5: 74%	SIMD 1 or 2: 26% SIMD 3–5: 74%	SIMD 1 or 2: 27% SIMD 3–5: 73%	0.906
Glucose Management Indicator (GMI) (mmol/mol)	61 (55–70)	61 (55–69)	64 (56–73)	0.258
Mean glucose (mM)	10.2 (9.0–12.2)	10.2 (9.0–12.0)	10.8 (9.2–12.8)	0.258
Time above range (%)	49 (35–66)	48 (35–64)	54 (36–74)	0.213
Time in range (%)	48 (33–62)	48 (35–62)	43 (27–61)	0.204
Time below range (%)	1 (0–4)	1 (1–4)	1 (0–3)	0.443
CV glucose (%)	36.4 (33.1–41.1)	36.4 (33.1–41.3)	36.5 (33.4–40.6)	0.891

^a^ Baseline CGM data available in 310/353 in the total cohort.

### 3.2 AID system settings

Median insulin delivered as basal was 57% (50–63) and, correspondingly, 43% (37–50) was delivered as bolus. Median total daily insulin dose was 41 units (30–54) or 0.52 units/kg (0.43–0.63). The median number of boluses delivered per day was 4.9 (3.9–6.4). Time in auto mode was 99% (94–100) of which time in full auto mode was 96% (89–98) and time in limited auto mode was 2% (1–4). About 42% of users selected active insulin time lower than 4 hours. A total of 61% of users selected the lowest possible glucose target (6.1 mM), with 17% selecting 6.7 mM, 13% selecting 7.2 mM and the remaining 9% selecting higher targets. Median insulin sensitivity (correction) factor was 2.8 (2.0–3.0) and median insulin to carbohydrate ratio was 1:9.3 (1:8.0–1:10.0).

### 3.3 Change in CGM metrics and consensus targets

**FIGURE 1:**
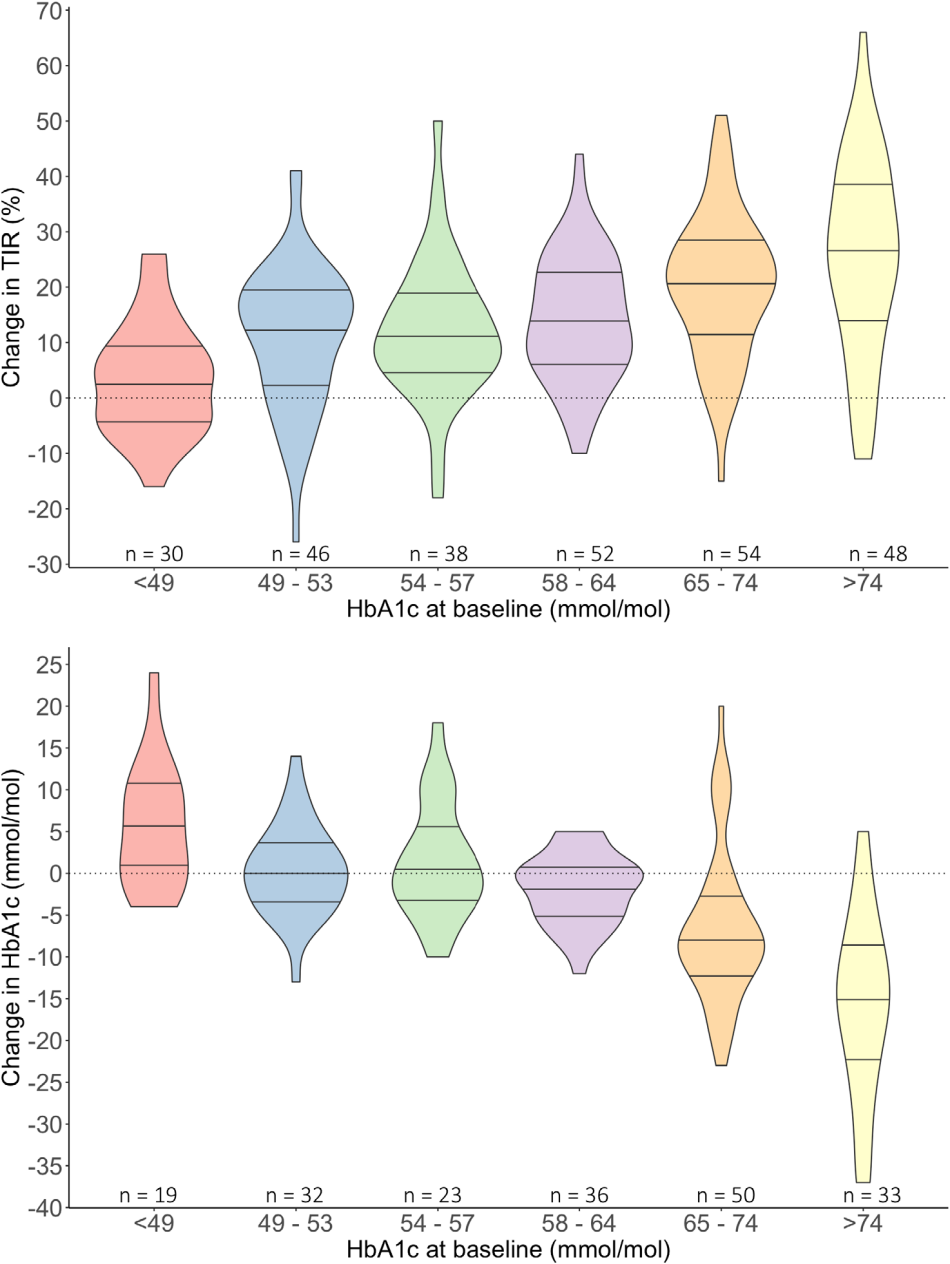
Change in TIR and HbA1c at final follow‐up stratified by baseline HbA1c. *p* < 0.001 for TIR and HbA1c.

**FIGURE 2:**
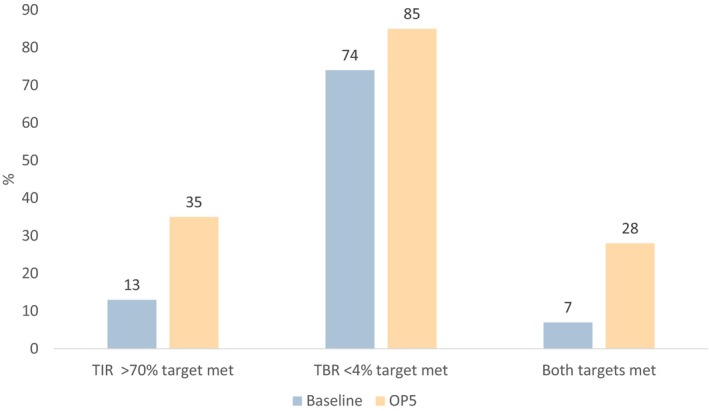
Change in those meeting consensus guidance targets for TIR, TBR, and both targets after commencement of AID. *p* < 0.001 for all comparisons.

Change in CGM metrics following Omnipod 5 are summarized in Table 2. Median difference in time in range was +16% (5–25), *p* < 0.001. Change in TIR was strongly influenced by baseline HbA1c (Figure 1) and was +27% (14–39) in those with HbA1c > 74 mmol/mol compared to +4% (−4–9) in those with HbA1c <49 mmol/mol (*p* < 0.001). The percentage of individuals meeting the >70% TIR target increased from 13% to 35% (*p* < 0.001) and those meeting the <4% TBR target also increased (74% to 85%, *p* < 0.001) (Figure 2). TIR increased by 26% [17–33] in people on MDI at baseline with HbA1c ≥ 58 mmol/mol (*p* < 0.001, *n* = 50).

**TABLE 2:** Changes in CGM metrics from baseline to final follow‐up (median duration 191 days [135–452]) in the total cohort and by CGM sensor system used.

	Total (*n* = 268)			Libre 2 (*n* = 191)			Dexcom G6 (*n* = 77)		
	Pre	Post	*p*	Pre	Post	*p*	Pre	Post	*p*
Mean glucose (mM)	10.2 (9.0–12)	9.0 (8.3–10.0)	<0.001	10.0 (8.9–11.7)	8.8 (8.2–9.7)	<0.001	10.8 (9.3–12.6)	9.6 (9.0–10.5)	<0.001
Glucose Management Indicator (GMI) (mmol/mol)	61 (55–69)	55 (52–60)	<0.001	60 (55–68)	54 (51–58)	<0.001	63 (56–72)	58 (55–62)	<0.001
Time above range (%)^a^	48 (35–64)	32 (23–42)	<0.001	46 (33–63)	29 (22–38)	<0.001	52 (38–66)	40 (31–48)	<0.001
Glucose > 13.9 mM (%)	17 (8–30)	8 (4–14)	<0.001	16 (8–29)	7 (3–12)	<0.001	20 (11–37)	11 (7–19)	<0.001
Glucose 10.1–13.9 mM (%)^a^	28 (21–32)	22 (18–27)	<0.001	28 (21–32)	21 (17–26)	<0.001	28 (22–31)	27 (22–31)	0.084
Time in range (%)^a^	48 (35–62)	66 (57–74)	<0.001	51 (36–63)	68 (61–76)	<0.001	46 (32–61)	59 (52–69)	<0.001
Time below range (%)^a^	2.5 (3.0)	1.9 (1.9)	0.007	2.6 (3.1)	2.2 (2.0)	0.282	2.2 (2.6)	1.1 (1.5)	0.001
Glucose 3.0–3.8 mM (%)^a^	1.9 (2.4)	1.6 (1.5)	0.150	2.0 (2.5)	1.9 (1.5)	0.731	1.7 (1.8)	0.9 (1.2)	<0.001
Glucose < 3.0 mM (%)	0.6 (1.5)	0.3 (0.6)	<0.001	0.7 (1.5)	0.3 (0.6)	<0.001	0.5 (1.6)	0.2 (0.5)	<0.001
CV glucose (%)	36 (33–41)	34 (31–38)	<0.001	36 (33–41)	34 (31–38)	<0.001	36 (33–41)	33 (31–37)	<0.001

*Note*: Low glucose metrics are reported as mean (SD).

^a^ Indicates significant differences in metric Δ (pre‐ and post‐AID) between sensor types (Libre 2 and Dexcom G6), all *p* < 0.05.

### 3.4 Change in HbA1c

Median change in HbA1c was −3 mmol/mol (−9–2) at median follow‐up of 231 (125–384) days after Omnipod 5 commencement (*n* = 193). Change in HbA1c was also significantly influenced by baseline HbA1c: −7 mmol/mol (−13 to −1) in those with HbA1c ≥ 58 mmol/mol vs. +1 mmol/mol (−3–2) in those with HbA1c <58 mmol/mol (*p* < 0.001). Change in HbA1c by baseline HbA1c category is presented in Figure 1. Interval from Omnipod 5 commencement to subsequent HbA1c was weakly inversely correlated (*R* −0.145, *p* = 0.043). Change in HbA1c was the same in people where paired CGM data were available (−3 mmol/mol [−9–2]) and in those with no paired CGM data (−3 mmol/mol [−9–0], *p* = 0.698).

### 3.5 Comparison of sensors

**FIGURE 3:**
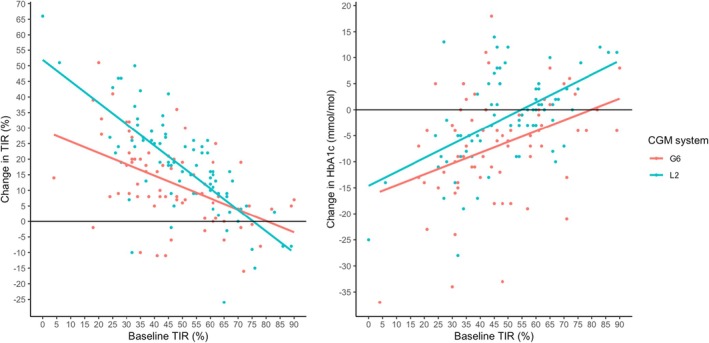
Relationship between baseline TIR and subsequent change in TIR and HbA1c following AID commencement (comparison of relationship by CGM system used).

Median time in auto mode was higher with Libre 2 (100% [97–100]) compared to Dexcom G6 (97% [90–99], *p* < 0.001). This was also true of full auto mode: 96% (91–98) vs. 94% (84–98), *p* < 0.001. There was no difference in time spent in limited auto mode: 2% (1–4) with Libre 2 vs. 2% (1–4) with Dexcom G6, *p* = 0.629. Time in manual mode was higher with Dexcom G6 (3% [1 – 10]) compared to Libre 2 (0% [0–3]), *p* < 0.001. Changes in CGM metrics comparing both sensors are presented in Table 1. There appeared to be different relationships between baseline TIR and change in TIR when considering each sensor (Figure 3). In total, there was a strong inverse correlation between baseline TIR and subsequent change in TIR (*R* −0.592, *p* < 0.001); however, this relationship was stronger with respect to Libre 2 (*R* −0.737, *p* < 0.001) compared to Dexcom G6 (*R* −0.539, *p* < 0.001) (*p* = 0.021 comparing correlation coefficients). There was also a strong relationship between baseline TIR and subsequent change in HbA1 (*R* 0.446, *p* < 0.001) but this did not differ significantly between sensor type (Libre 2: 0.491, *p* < 0.001 and Dexcom G6 *R* 0.365, *p* = 0.002; *p* = 0.352 comparing correlation coefficients) (Figure 3). Improvements in TIR (*p* = 0.033) and TAR (*p* = 0.038) were greater in the Libre 2 cohort, and TBR improvements were greater in the Dexcom G6 cohort (*p* = 0.016) (Table 2). Baseline TIR (*p* = 0.124), TAR (*p* = 0.104) and TBR (*p* = 0.327) were not significantly different between the Dexcom G6 and Libre 2 cohorts.

### 3.6 Predictors of improvement in TIR and HbA1c

**FIGURE 4:**
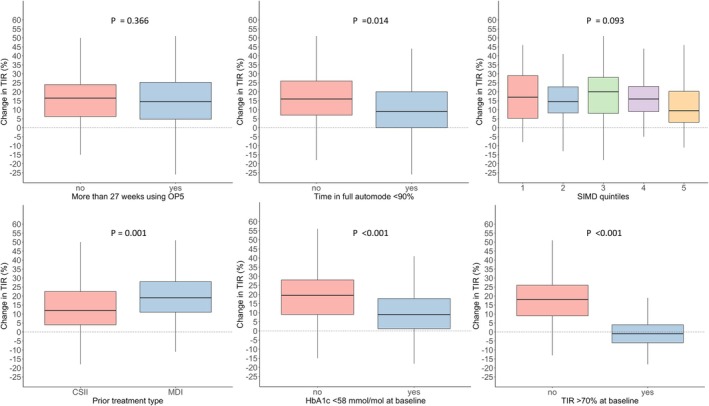
Change in TIR by duration of AID use, time in full automode, SIMD quintile, prior treatment type, baseline HbA1c and baseline TIR.

In addition to higher baseline HbA1c and lower baseline TIR (Figures 1 and 3), only a few additional metrics were associated with TIR response to Omnipod 5. Those on multiple daily injections (MDI) at baseline had a greater TIR response on Omnipod 5 (19% [11–28] vs. 12% [4–23], *p* = 0.001) (Figure 4). Those who were in full auto mode for less than 90% of the time had a lower TIR response (9% [0–20] vs. 16% [7–26], *p* = 0.014). Duration of Omnipod 5 use was not associated with TIR response (*p* = 0.366, Figure 4) and the correlation between days of Omnipod 5 use and change in TIR was not statistically significant (*R* −0.110, *p* = 0.072). Similarly, SIMD quintile was not associated with TIR response (*p* = 0.093, Figure 4).

In logistic regression analysis, the following were independently associated with a TIR increase of at least 5%: baseline TIR (OR 0.83 per % [0.78–0.87], *p* < 0.001); percentage insulin delivered as bolus (OR 1.16 per % [1.10–1.24], *p* < 0.001); lowest glucose target (6.1 mM) set on Omnipod 5 (OR 5.4 [2.1–15.3], *p* < 0.001); full automated mode used <90% of the time (OR 0.24 [0.09–0.59], *p* = 0.002); total daily insulin dose (OR 0.95 per unit [0.92–0.98], *p* = 0.004); and BMI (OR 1.14 per unit BMI [1.03–1.29], *p* = 0.022). The following were independently associated with a fall in HbA1c of at least 5 mmol/mol: baseline HbA1c (OR 1.24 per mmol/mol [1.34–1.67], *p* < 0.001); full automated mode used <90% of the time (OR 0.23 [0.13–0.88], *p* = 0.032); and active insulin time set lower than 4 h (OR 2.6 [1.05–7.1], *p* = 0.045). CGM system was not an independent predictor of response with respect to either TIR or HbA1c.

### 3.7 Weight

Median weight prior to AID commencement was 74.8 kg (66.0–88.0) and rose to 76.8 kg (66.6–88.8), *p* = 0.002. Change in HbA1c following AID was inversely correlated with weight gain (*R* −0.174, *p* = 0.024).

## 4 DISCUSSION

In a real‐world context, Omnipod 5 was associated with substantial and durable improvement in TIR, in concert with significant reduction in TBR. Omnipod 5 was also associated with significant reduction in HbA1c, which was most marked in those with elevated HbA1c at baseline. Individuals with higher HbA1c (lower TIR) are frequently under‐represented in clinical trials of AID,^5^ with implications for potential underestimation of the effect of therapy and also generalizability of results. In line with this, the TIR increase of 16% in our cohort was larger than that observed in the Omnipod 5 pivotal study (9.3% in adults).^6^ Interestingly, our HbA1c change in those with HbA1c ≥58 mmol/mol on prior MDI was identical (−9 mmol/mol) to the recently reported RADIANT randomized controlled trial^11^ of Omnipod 5. We noted modest weight gain following AID commencement, which was only weakly associated with improvement in HbA1c.

The clearest predictor of improved TIR (and HbA1c) was prior low TIR (high HbA1c) and this is in line with prior AID studies focused on individuals with high baseline HbA1c.^12^, ^13^ Taken together, these data present a compelling argument for prioritizing AID for those at greatest risk of complications, who stand to achieve the largest improvements in glucose metrics, and by extension, the greatest reduction in risk of complications.^14^ Lower socioeconomic status is a strong predictor of high HbA1c and is also associated with substantially lower CSII use.^15^ Improvements in glycaemic metrics with AID appear to be independent of socioeconomic status and may help address prior inequalities in outcome related to numeracy skills.^16^ Another insight from our cohort relates to the importance of supporting maximum time in automode and is consistent with reports in other AID systems.^17^ In addition, setting the lowest glucose target, considering shorter active insulin time, and delivering more bolus insulin may all be associated with a greater likelihood of TIR benefit. However, the risk of hypoglycaemia and other factors must be considered when optimizing settings.

Interpretation of differences related to CGM sensors is complicated. We observed greater time in full automode in those using Libre 2 plus (compared to Dexcom G6) but, despite this being a predictive factor for greater improvement in TIR, sensor choice was not independently associated with a greater likelihood of TIR improvement. We previously reported that Dexcom G6 was more likely to be associated with reduction in TBR^18^ and this remained true in this larger cohort. Libre 2 plus appeared to be associated with greater increases in TIR. A substantial problem in interpreting these differences is the lack of standardization between CGM systems,^19^, ^20^, ^21^ which must surely account for the discordance in the relationship between baseline TIR and AID‐related change between sensors in our cohort. Very small between‐sensor differences in measurement of glucose at the lower and upper end of the target range have the potential to result in substantial differences in TIR. We would suggest that subsequent prospective studies of AID should report outcomes using more than one CGM system until adequate standardization is achieved.

We feel the results from this cohort are highly generalizable to the adult population of people living with type 1 diabetes. Paired CGM data were available in 76% of Omnipod 5 users, and although younger people were slightly under‐represented, there was no difference in HbA1c outcomes between those with paired CGM data and those where this was not available. In addition, age was not noted to be a factor predictive of glycaemic outcomes in Omnipod 5 users. Although the variability in follow‐up duration could be regarded as a methodological weakness, this permitted the opportunity to assess the influence of time using AID as a predictor of outcomes. To that end, there was no significant influence of Omnipod 5 use duration upon change in TIR, and the impact on HbA1c was negligible (with a weak trend towards greater reduction over time), suggesting a durable effect upon improvements in glycaemic management.

## 5 CONCLUSIONS

Omnipod 5 is associated with significant improvements across all CGM metrics and HbA1c in a real‐world clinical context. Benefits are greatest in those with the highest HbA1c at baseline. Differences in CGM metrics between sensor systems suggest a need for greater standardization of interstitial glucose measurement.

## CONFLICT OF INTEREST STATEMENT

FWG has received speaker fees from Abbott, Dexcom, and Insulet. SF collaborates with and receives funding from Novo Nordisk Cell Therapy Programme.
